# Characterization and Robust Classification of EEG Signal from Image RSVP Events with Independent Time-Frequency Features

**DOI:** 10.1371/journal.pone.0044464

**Published:** 2012-09-18

**Authors:** Jia Meng, Lenis Mauricio Meriño, Nima Bigdely Shamlo, Scott Makeig, Kay Robbins, Yufei Huang

**Affiliations:** 1 Picower Institute for Learning and Memory, Massachusetts Institute of Technology, Cambridge, Massachusetts, United States of America; 2 Stanley Center for Psychiatric Research, Broad Institute, Cambridge, Massachusetts, United States of America; 3 Department of Electrical and Computer Engineering, University of Texas at San Antonio, San Antonio, Texas, United States of America; 4 Swartz Center for Computational Neuroscience, Institute for Neural Computation, University of California San Diego, La Jolla, California, United States of America; 5 Department of Computer Science, University of Texas at San Antonio, San Antonio, Texas, United States of America; 6 Department of Biostatistics, University of Texas Health Science Center at San Antonio, San Antonio, Texas, United States of America; University of British Columbia, Canada

## Abstract

This paper considers the problem of automatic characterization and detection of target images in a rapid serial visual presentation (RSVP) task based on EEG data. A novel method that aims to identify single-trial event-related potentials (ERPs) in time-frequency is proposed, and a robust classifier with feature clustering is developed to better utilize the correlated ERP features. The method is applied to EEG recordings of a RSVP experiment with multiple sessions and subjects.

The results show that the target image events are mainly characterized by 3 distinct patterns in the time-frequency domain, i.e., a theta band (4.3 Hz) power boosting 300–700 ms after the target image onset, an alpha band (12 Hz) power boosting 500–1000 ms after the stimulus onset, and a delta band (2 Hz) power boosting after 500 ms. The most discriminant time-frequency features are power boosting and are relatively consistent among multiple sessions and subjects.

Since the original discriminant time-frequency features are highly correlated, we constructed the uncorrelated features using hierarchical clustering for better classification of target and non-target images. With feature clustering, performance (area under ROC) improved from 0.85 to 0.89 on within-session tests, and from 0.76 to 0.84 on cross-subject tests. The constructed uncorrelated features were more robust than the original discriminant features and corresponded to a number of local regions on the time-frequency plane.

**Availability:** The data and code are available at: http://compgenomics.cbi.utsa.edu/rsvp/index.html

## Introduction

A brain computer interface (BCI) system allows human subjects to use their brains to directly communicate with or control an external device [1]. One possible application of BCI is to search for the target images from a large collection of seemingly undesirable ones. Traditional human-based target image recognition is extremely laborious, slow, and inconsistent; it becomes increasingly infeasible for a prolonged processing of large image collections. Computer-based image recognition often suffers from low classification accuracy. In contrast, the BCI-based systems may overcome the respective shortcomings of a human-based or a computer-based system with the potential to provide more efficient and effective classification.

One BCI system for image classification is realized with the rapid serial visual presentation (RSVP) of images [2]–[4]. RSVP [5] specifies a process, during which the images (or text, video) are displayed one-by-one in a fixed focal position. A small set of target images of interest are embedded among the presented images, and Electroencephalogram (EEG) recording of subjects' brain activities are collected through the experiment.

### Characterization of event-specific signatures

As a first step, a successful BCI system needs to extract the event-specific signatures that characterize the brain signals specific to the target (or non-target) images embedded in the EEG recordings. This is often achieved with a training process, in which the signatures are extracted from training data whose event-association are already known. From image RSVP, one such signature is the P300 event-related potentials (ERPs), which is a positive potential that can be observed approximately 250–900 ms after the stimulus onset and is most frequently elicited in an oddball paradigm [6], in which rare target events are interspersed with frequent non-target events. Since the amplitude of a typical ERP is on the order of 1 to 10 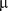
V, while the background EEG amplitude is on the order of 100 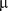
V, a high performance BCI system with robust ERP identification is extremely challenging due to the low signal-to-noise ratio. A major limitation of many current approaches is that they mostly fail to explicitly address the event-specific frequency domain signatures, which have been shown to be more effective than the time domain signatures [3]. Previously, connections have been established between frequency bands and brain activity including, for instance, the association of gamma band with cross-modal sensory processing [7] and the relationship between theta band and inhibition of elicited responses [8]. Revealing the temporal-frequency-spatial characteristics of the discriminant features and the underlying spectral responses related to the image RSVP task may provide greater insight into the brain functions responding to the task, thus enabling better understanding of human cognition.

### Classification of unknown recordings

A successful BCI system also needs to effectively utilize the event-specific signatures for classification of EEG recordings whose event-association is unknown. This concerns building an efficient and robust classifier. So far, a number of classifiers have been implemented for classification of RSVP tasks, including logistic regression and Fisher's linear discriminant analysis (LDA) [3], [9]. The high dimensional and highly correlated discriminant features are difficult for conventional classifiers, which often assume feature independence and are constructed for a relatively smaller number of features. It has been pointed out [10] that removal of the correlation between features can significantly improve the classification performance.

In this paper, we conducted a time-frequency analysis of the EEG recordings during the RSVP paradigm and systematically identified the discriminant time-frequency features of target events and non-target events with their statistical significance. A visualization system is developed to illustrate the space, time and frequency distribution of the discriminant features. Moreover, a cluster-based LDA classifier is implemented to classify target and non-target images. Result shows that, after combining the correlated features using hierarchical clustering with the cluster medians as new features, the cluster based LDA classifier is capable of incorporating more information than traditional feature-based LDA methods and performs much better in terms of Az score (the area under the receiver operator characteristic curve).

## Materials and Methods

### Experiment design and data preprocessing

The RSVP EEG recordings were obtained from [3]. Participants are presented a series of bursts of small image clips in a RSVP paradigm. Each burst lasts for 4.1 s and consists of 49 image clips presented at a speed of 12 clips/second. Each burst may contain one target image, which shows an airplane that is not present in other non-target images. To ensure no interference from burst edges, the target clip is only presented after 500 ms from the onset and before 500 ms from the offset of the burst. EEG recordings were collected using BIOSEMI active view 2 system with 256 electrodes at 256 Hz sampling rate with 24-bit digitization. (Please refer to [3] for more details about the experimental design and data acquisition method.) The data set we adopted consists of 10 EEG recording sessions from 5 subjects (2 sessions per subjects).

Data preprocessing was conducted using EEGLAB [11] under the MATLAB environment. The frequency domain filtering was performed by applying 3 independent IIR Butterworth filters of order 3 including an IIR high-pass filter (2 Hz), an IIR low-pass filter (50 Hz), and an IIR band-rejection filter (40–80 Hz). Here, the high-pass filter (2 Hz) is mainly used to filter out slow artifacts, such as electrogalvanic signals and movement artifacts; while the low-pass filter (50 Hz) eliminates the high-frequency artifacts, such as electromyographic signals. The additional notch filter compensates for artifact noise caused by electrical power lines (60 Hz in the United States) [12].

### Time-frequency feature calculation and normalization

**Figure 1:**
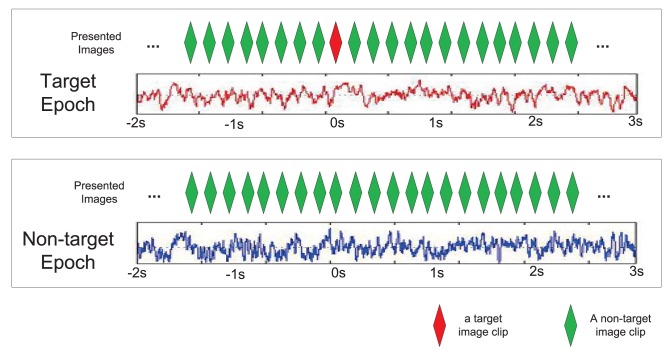
Target and non-target epochs. A target epoch consists of EEG recordings 2 s before the target image clip onset until 3 s after its onset, during which there is only one target image presented. No target image clip's presented in a non-target epoch.

The target and non-target epochs were first isolated. As shown in Fig. 1, a target epoch contains the EEG recordings from 2 s before to 3 s after the onset of a target image that has been correctly identified; similarly, a non-target epoch contains the EEG recordings from 2 s before to 3 s after the onset of a non-target image that has also been correctly identified. With the assumption that the target or non-target image is present at 0 s of the epoch, each epoch records the EEG signal from −2 s to 3 s. The raw EEG data are two dimensional electrical potentials in the space-time domain, where the spatio information naturally resides in the EEG recordings as the locations of the EEG electrodes on the skull. To capture the frequency characteristics, the Gabor wavelet transformation was applied to each individual channel data separately over all epochs at frequencies [2.0 2.6 3.3 4.3 5.6 7.2 9.3 12.0 15.5 20] Hz within [−2 s, 3 s] with 256 Hz sampling rate. The time-frequency transformed data were also down-sampled by 16 Hz to reduce sample dependence. The final transformed data of each epoch represent the power of EEG recording distributed in 3 dimensions including the space (channel), the time, and the frequency dimension.

To eliminate cross-epoch variations, a log transformation of power was applied, and the power distribution over sampling time at each channel/frequency was then normalized based on the information before 0 s of the epoch. Specifically, let 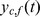 represent the log-power of channel 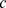 at frequency 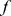 at time 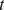. Then for 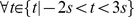, the normalized power 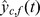 is calculated as
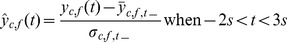
(1)where 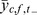 and 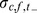 are the mean and the standard deviation of {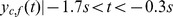}. Here, the constraint 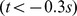 is introduced to ensure that the normalization is independent of different event types (target or non-target image), for the power of time-frequency analysis close to time 0 can be affected by the specific event types at time 0; on the other hand, the constraint 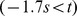 is introduced to ensure that the power is not affected by the onset of the epoch in time-frequency analysis. For convenience, we refer to the normalized log-power as simply the power in the following.

### Identification of Discriminant ERP Features

The discriminant time-frequency features are defined as the power distribution in specific space, time, and frequency regions that are distinct in target and non-target events. This features, sometimes called event-related spectral perturbation (ERSP) [13], are 3-dimensional corresponding to channel, frequency and time, respectively.

Let 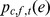 define the power at channel 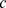, time 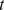, and frequency 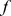 of epoch 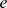, and 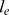 be the event type of the 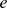-th epoch, with 1 representing the target event and 0 the non-target event. Then, the goal of identifying discriminant ERPs is to determine the 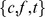 triples such that 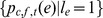 and 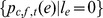 are significantly different. Note that these discriminant 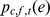 can be used as the features to classify the target epoch from non-target epoch. The significance of a single discriminant 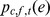 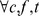 is evaluated by its discriminant power defined as the area under receiver operating characteristic (Az score) of an LDA classifier.

Specifically, for this case, the LDA assumes that the probability density functions (PDFs) of 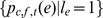 and 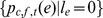 are both normally distributed but with different mean and the same variance parameters, or 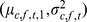 and 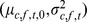, respectively. Then, for some threshold constant 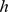, the decision criterion becomes,
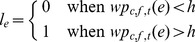
where 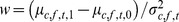. For a binary classifier system, the ROC curve is a graphical plot of the sensitivity vs 1-specificity as a discrimination threshold 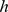 is varied. The area under ROC, which is equal to the probability that a classifier will rank a randomly chosen positive event higher than a randomly chosen negative one [14], has been traditionally used for classifier performance evaluation.

### Classification based on uncorrelated features

Classifiers including LDA have been applied to the RSVP classification problem and achieve considerable success [3]. However, one limitation of LDA is that it cannot handle large number of features efficiently, and a feature selection process is usually needed. Since the number of possible feature combinations increases exponentially with the number of features, an exhaustive search of the optimal combination is usually infeasible. Suboptimal yet accurate feature selection algorithms include the filter and the wrapper approaches, where the wrapper approaches have been shown to provide better performance. The popular sequential forward search wrapper method relies on a greedy search, where the features are first ranked decreasingly according to their discriminant power, or Az score, and then the top 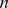 features that lead to the best LDA classification performance are retained as the optimal feature set. This approach assumes that the features are independent. When the features are correlated, it may not perform well [10].

Given that the correlation between space time-frequency features can be high, a direct application of the sequential forward search is less favorable. Instead, we seek to derive uncorrelated features before the feature selection. To this end, hierarchical clustering was applied to group the correlated features and the cluster centroids were extracted as the independent features [15]. For simplicity, we call this method “cLDA”. One issue with cLDA is the selection of the number of clusters, which will be discussed in the results section.

## Results

### RSVP target events are characterized mostly by power activation in time frequency domain

After Az scores for all 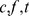 features were calculated, the features were classified into 2 categories, the activated features that have a power boost in target events vs. nontarget event, and the repressed features that have a power decrease in the target events.

**Figure 2:**
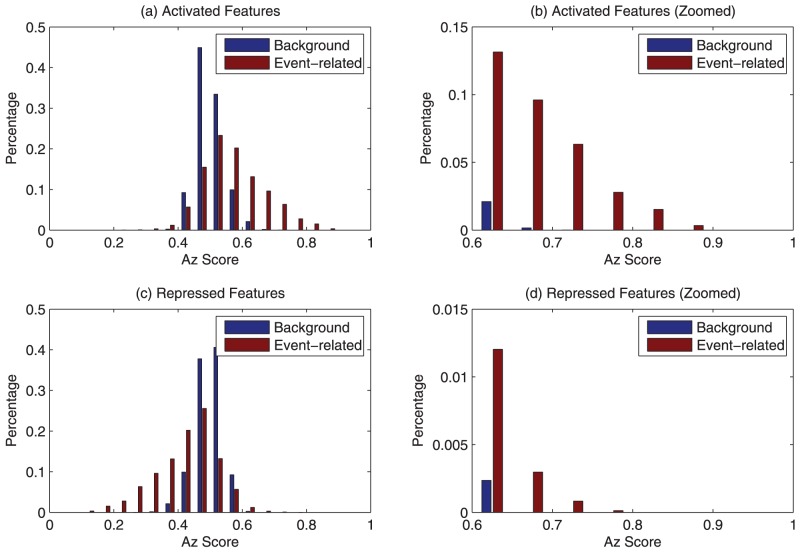
The histogram of the Az scores. With the Az scores of features during −1.7 s to −0.3 s used as background, it can be clearly seen from the figure that a significant number of time-frequency features have a power boost in the target events as compared to the non-target events ((a) and (b)), while the number of repressed time-frequency features are relatively small ((c) and (d)).

The features in each category were further divided into two periods, i.e., the background period (−1.7 s to −0.3 s) and the event-related period (0 s to 2.7 s). Since the background period is before the stimulus onset, the features in background are less discriminant and should be randomly distributed. In contrast, since the ERP responses are in the event-related period, the features in this period should be more discriminant It can been seen from Fig. 2 that the event-related period clearly has larger discriminant power than the background, and the features with large discriminant power are mostly activated features.

### Patterns characterizing RSVP target events in time frequency domain

**Figure 3:**
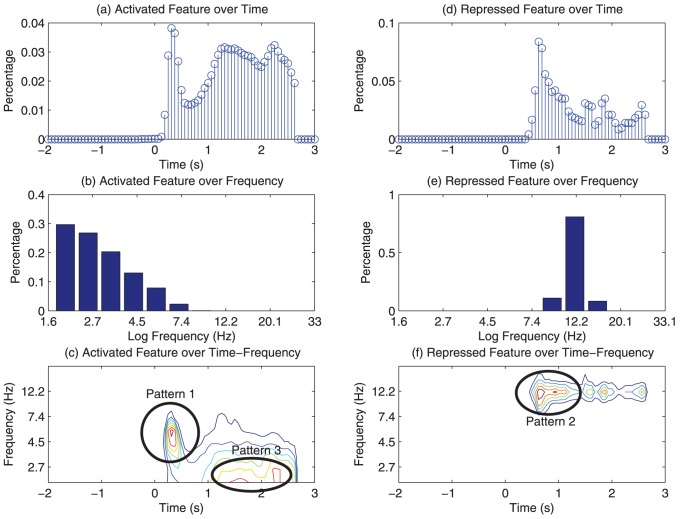
Time-frequency distribution of discriminant features of session 1, subject 1. Both the activated (a) and the repressed (d) discriminant features appear after 0 s (or stimulus onset). The activated features are mostly lower than 8 Hz (b), while the repressed features are centered around 12 Hz (e). Compared with the non-target events, the target events are mainly characterized by 3 distinct patterns in time-frequency domain, i.e., a power boost at around 4.3 Hz 300–500 ms after the target image onset (Pattern 1 in (c)), a power repression at 12 Hz during 500–1000 ms (Pattern 2 in (f)), and a power boost at 2 Hz after 500 ms (Pattern 3 in (c)).

To gain insights into the discriminant features, we plotted the distribution of the most significantly discriminant features in time, frequency, and time-frequency dimensions (Fig. 3). Here we are only interested in the features, whose Az scores are larger than those of any background features and are statistically significant. To assess the statistical significance, we used Az scores of the background features to construct an empirical distribution for the non-discriminant features. Since the background features are extracted from EEG recordings before the target stimulus, they are guaranteed to be non-discriminant. As a result, a feature's Az score will have a 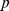-value of 0.05 if it is larger than 95% of the background features' Az scores. The plotted features here are discriminant features with a 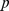-value smaller than 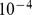. It can been seen from Fig. 3-(a,b,c) that most of the activated features appear in relatively low frequency band after target image onset, while the repressed features are centered around 12 Hz and last between 500–1000 ms after the target image onset (Fig. 3-(d,e,f)).

Examination of the discriminant features in time-frequency dimension indicates that, compared with non-target events, the target events are mainly characterized by 3 distinct patterns in time-frequency domain (Fig. 3-(c,f)), i.e., a power boost at around 4.3 Hz 300–700 ms after the target image onset (Pattern 1), a power decrease at 12 Hz during 500–1000 ms (Pattern 2), and a power boost at 2 Hz after 500 ms (Pattern 3).

### Classification by cLDA based on uncorrelated features

We investigated performance of the proposed cLDA. First, we examined the impact of the number of clusters on the performance of cLDA. To ensure the cluster centers, or c-features, used by cLDA are discriminant, the individual features are first ranked in increasing order of Az score and the clustering is applied to a certain number of top ranked features. We tested cLDA with different numbers of top features 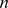 including 100, 200, 400, 800, 1600, 3200, 6400, 12800, 25600, 51200 (increased exponentially).

**Figure 4:**
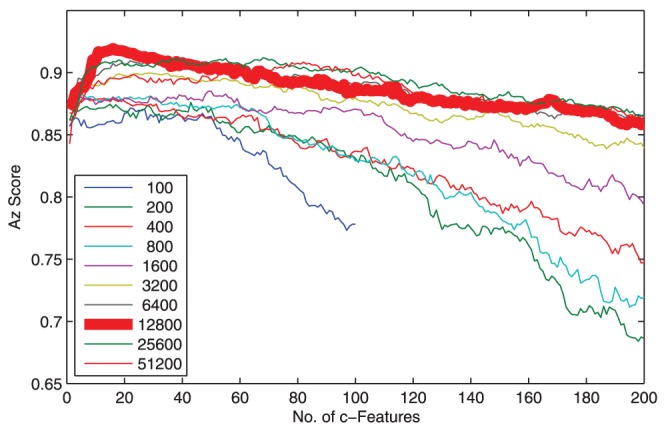
Plot of Az score vs the number of clusters. The performance of cLDA is robust against the number of features. cLDA classification performance is slightly affected by the number of features. As the number of top features increases, the classification performance first increases then decreases. Given a specific number of features, the optimal number of clusters is usually between 10 to 100. The best result is achieved at using 20 clusters obtained from 12800 top features.

To obtain a satisfactory performance, the total number of selected features, 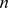, needs to be big enough to incorporate a sufficient number of discriminant features but stringent enough to exclude the non-informative time-frequency features. For each 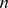, we evaluated Az scores as a function of the number of clusters 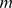 or the number of c-features. It can been seen from Fig. 4 that, cLDA classification performance is affected by the number of selected top features: As the number of individual top features 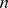 increases, the classification performance first increases then decreases. For the tested session (session 1 of subject 1), the best performance is achieved with 12800 features clustered into 20 clusters. The best classification performances are summarized in Table 1. In general, the classification performance of cLDA is rather robust against the number of top features. The largest performance difference is about 0.05 between cLDA based on 12800 and 800 top features.

**Table 1:** Performance of cLDA with different number of top features.

Feature No.	100	200	400	800	1600	3200	6400	12800	25600	51200
c-Feature No.	25	12	13	14	48	26	30	20	62	82
Az Score	0.867	0.871	0.878	0.879	0.881	0.899	0.909	**0.916**	**0.909**	**0.906**

The performance of cLDA is robust against the number of selected top features.

**Figure 5:**
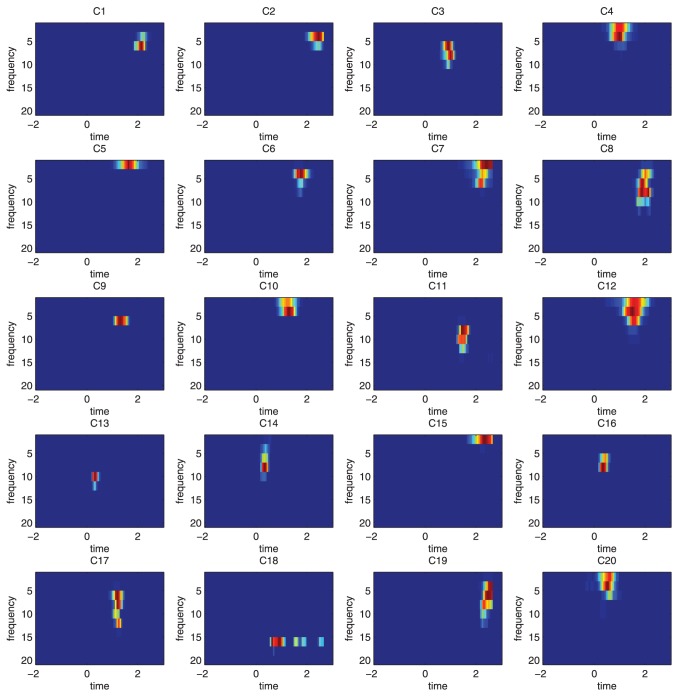
Display of features of the same cluster. cLDA achieves its best performance with top 12800 discriminant time-frequency features grouped into 20 clusters. The features within the same cluster are projected into time-frequency domain and the distribution of the features in time and frequency for the 20 clusters are depicted in the 20 sub-figures. As is shown in this figure, the correlated features clusteredwere localized together within the time-frequency space.

To analyze the features within a feature cluster and gain insights into cLDA, the features within one cluster were projected into the time-frequency space and depicted in Fig. 5. It can been seen that most of the features within the same cluster are located within a relatively tight time-frequency range. The c-features of cLDA can be considered as the exemplars of a group of discriminant features that are highly correlated with each other and located within the same region in the time frequency domain.

### Comparison of cLDA and LDA on multiple sessions

The classification performance of LDA and cLDA are tested on 10 individual RSVP sessions and compared in term of Az score. For each session, both classifiers are trained and tested by a 10-fold cross-validation on 10 individual sessions from 5 subjects (2 EEG recording sessions from each subject). In this test, the number of top features of LDA can be any integer from 1 to 200, while cLDA combines the top 12800 features into 1 to 200 c-features. (Note that the computational complexity of cLDA based on 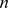 c-features is the same as LDA based on 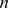 features.)

**Figure 6:**
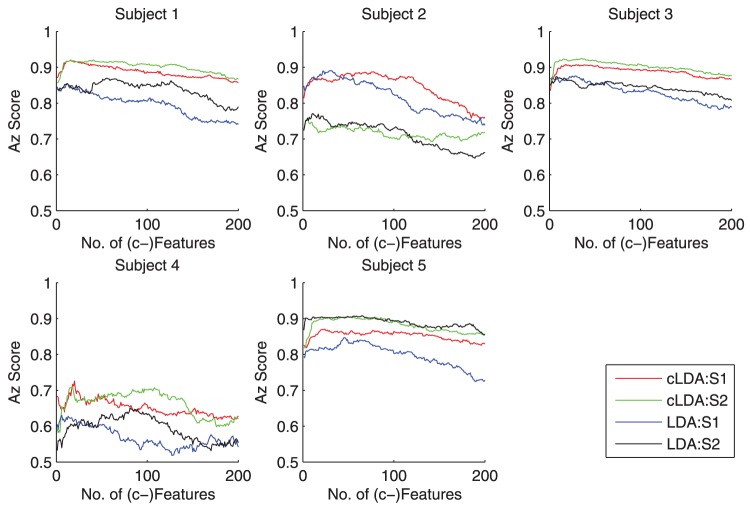
Comparison of cLDA and LDA on 5 subjects. cLDA outperforms LDA on 3 of the 5 tested subjects (1,3,4) and is with comparable performance on the remaining 2 subjects (2,5). There are two EEG sessions (S1, S2) from each participants.

**Figure 7:**
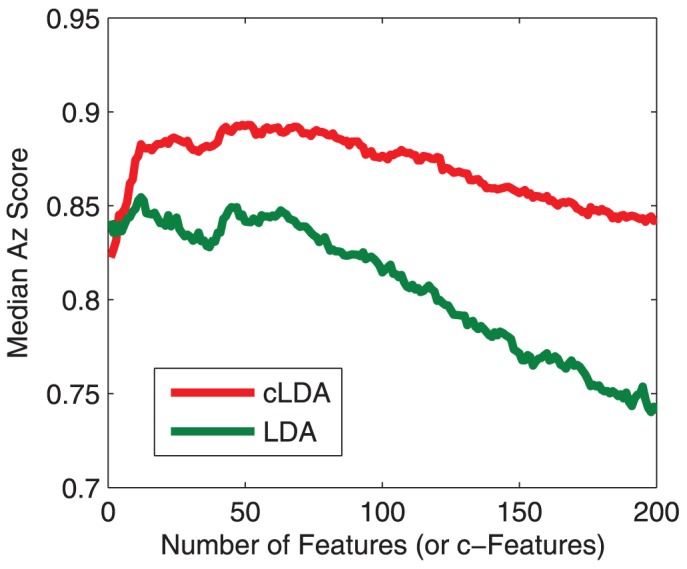
Median Az score of cLDA and LDA with difference number of (c-)features based on 10 tested sessions. cLDA consistently outperforms LDA, despite the number of (c-)features used.

The Az score of LDA and cLDA on different sessions/subjects as a function of the number features or c-features are shown in Fig. 6. It can be seen that, for the same number of features and c-features, cLDA outperforms LDA on three of the five tested subjects (1,3,4) and is comparable with LDA on the other two subjects (2,5). Next, choosing only the best performances for cLDA and LDA, we summarized the performance on each of tested session/subject in Table 2. The best performance of cLDA is better than that of LDA in 8 of the 10 tested sessions, which are consistent on three of the five tested subjects (with mixed performance on the rest two subjects). The median performance of cLDA vs LDA is (0.89 vs 0.85). Overall, the median Az scores for all 10 tested session clearly indicate that cLDA performs almost always better than LDA regardless the number of c-features used (Fig. 7)

**Table 2:** Comparison of LDA and cLDA on 10 EEG recording sessions from 5 subjects.

	S1∶1	S1∶2	S2∶1	S2∶2	S3∶1	S3∶2	S4∶1	S4∶2	S5∶1	S5∶2	Median
LDA	0.86	0.85	0.87	**0.77**	0.86	0.87	0.62	0.6	0.81	**0.9**	0.85
cLDA	**0.9**	**0.91**	**0.88**	0.73	**0.9**	**0.92**	**0.68**	**0.68**	**0.86**	0.9	**0.89**

S
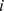
∶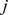 denotes session 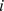 subject 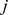.

cLDA returns the better results on 9 of the 10 tested sessions.

### Comparison of original features and c-features with SVM

To further investigate the advantages of the proposed c-features over the conventional time-frequency features, we tested their performance under the support vector machine (SVM) classifier [16].

**Figure 8:**
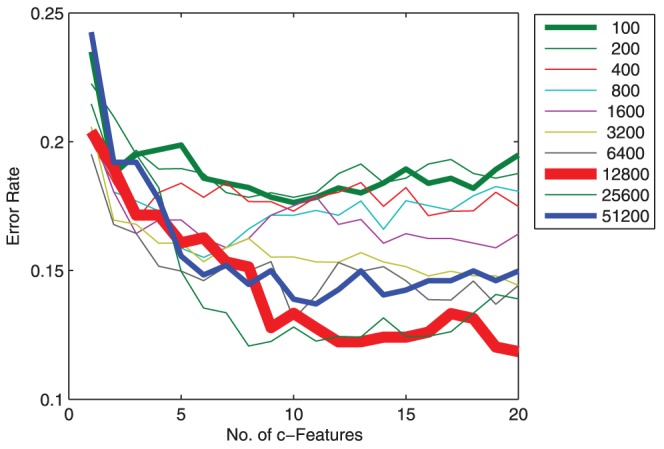
Selection of the optimal feature set. The figure shows the error rate of SVM vs. top ranked discriminant time-frequency features. Similar to the LDA classifier, the optimal set of original features are the top 12800 most discriminant features.

**Figure 9:**
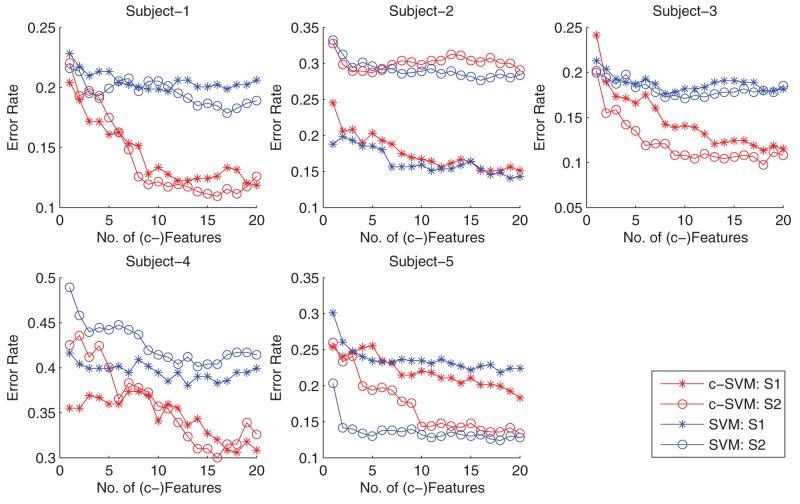
Comparison of SVM classifier based on c-features with that based on individual features. The figures show the error rate of classifiers vs. the number of c-features for five subjects, each with 2 sessions (S1 and S2). Overall, SVM with c-features shows clear improvement in error rate over that with individual features for three of the five tested subjects (1, 3, 4), and yields similar performance for the remaining two subjects (2, 5). This result is consistent with the LDA classifier.

In the first experiment, model selection was implemented to determine the best set of individual features, from which independent c-features were obtained. As shown in Fig. 8, the optimal feature set consists of the top 12800 most discriminant features. In the second experiment, the SVM classifier based on c-features was compared against that based on individual time-frequency features and the result is shown in Fig. 9. It can be seen from the figure that SVM with independent c-features clearly outperforms that with the individual features for three of the five tested subjects (1, 3, 4), and yields comparable results for the other two subjects (2 and 5). This result is consistent with result of the LDA classifier, once again demonstrating the advantage of the proposed c-features. The fact suggests that the advantages of our proposed c-features are general and can achieve improved performance with other classifiers.

### Robustness analysis of the discriminant features among multiple subjects

We also investigated the robustness of the discriminant c-features across different sessions, since the robustness of classification is important for improved performance for cross-session/subject test. It is necessary to first define the repeatability of a c-features:

- For an ERP feature, it is considered repeated if it appears in two EEG recording sessions if and only if its 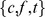 appears among the top discriminant features in both EEG sessions
- For a c-feature, since it is constructed from a cluster of ERP features, their repeatability is thus evaluated based on the original feature clusters. A feature cluster is considered repeated if and only if there is a significantly overlap between two feature clusters from two sessions. More specifically, if two feature clusters are significantly overlapped with each other with a 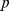-value (Fisher's exact test) smaller than 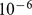, their corresponding c-features are considered repeated in both sessions. Here, the significance level 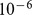 is calculated by 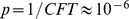, where 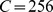 is the total number of channels, 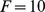 is total number of frequencies in time-frequency analysis, and 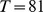 is the total number of samples in the 5 s epoch. This significance level is chosen to ensure the feature and c-feature have the same probability to appear by random in a different session.

**Figure 10:**
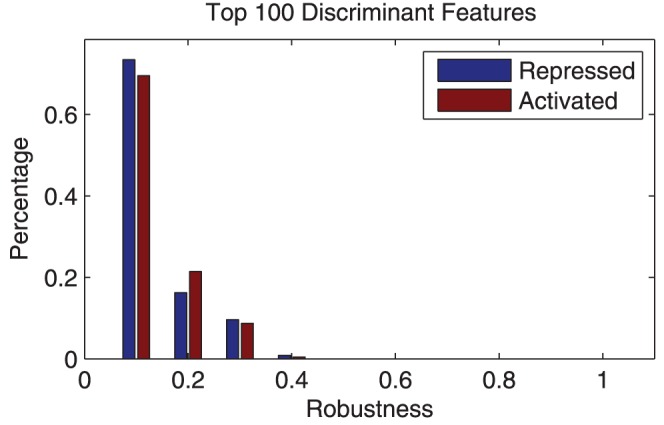
Robustness of the top 100 activated and repressed features. Features are considered robust on when power of the same channel, time, frequency appears in the top 100 most discriminant features. The top 100 discriminant features are not very robust across different sessions.

**Figure 11:**
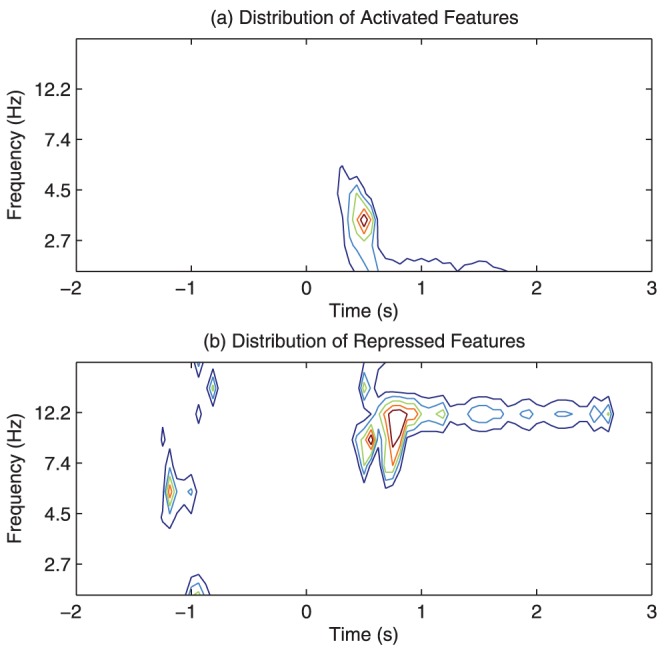
The time-frequency distribution of the top 100 most discriminant features among 10 tested sessions. The top 100 discriminant time-frequency features appear at locations corresponding to previously identified time-frequency patterns. i.e., a power boost at around 4.3 Hz 300–500 ms after the target image onset, a power decrease at 12 Hz during 500–1000 ms, and a power boost at 2 Hz after 500 ms. However, there are visible differences, indicating considerable variation of the top features among 10 sessions.

The robustness of c-features is then defined as the percentage of the 10 test sessions that a feature repeatedly appears. We first tested the robustness of the top 100 activated and repressed discriminant features. As shown in Fig. 10, the top 100 features are not significantly repeatable among multiple sessions. The distribution of these features in time-frequency as shown in Fig. 11 is relatively consistent with Fig. 3. The top 100 features do represent the 3 time-frequency patterns as shown in Table 3. However, significant variation also exist. This result demonstrate that the classifiers based on top features are not robust, i.e., the classifier trained in one session will not perform well in others sessions.

**Table 3:** The 3 groups of discriminant features in time-frequency domain.

Number	Time	Frequency	Type	Property
Pattern 1	0.3 s to 0.7 s	4.3 Hz	Theta band	Activation
Pattern 2	0.5 s to 1 s	12 Hz	Alpha band	Repression
Pattern 3	0.5 s to 2.5 s	2 Hz	Delta band	Activation

The 3 groups of features reside in different frequency bands and time periods.

**Figure 12:**
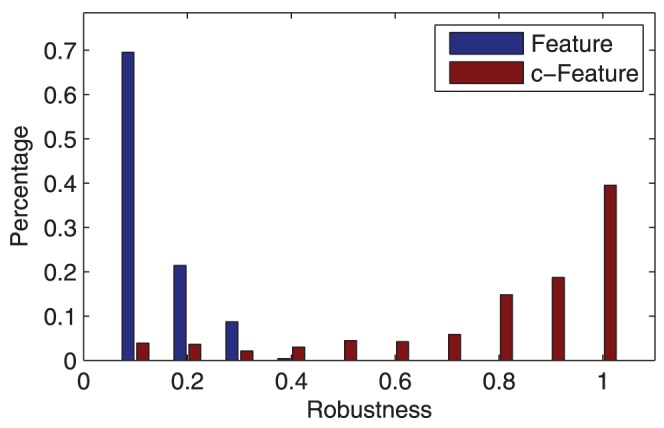
Comparison of robustness of features and feature clusters. Compared with top features, c-features are much more robust across different sessions. While more than 80% of the c-features appear in more than 1 of the 10 tested sessions, only around 31% of the top 100 features appear more than once. At the same time, none of the top features appears on more than 50% of the tested sessions as opposed to more 80% of the c-features.

As a comparison, we tested the robustness of c-features and compared it with that of the top features. The result is shown in Fig. 12. Compared with individual features, c-features are much more robust across different sessions. More than 80% of the c-features appear in more than 1 of the 10 tested sessions as opposed to only around 31% of the top 100 features. While none of the top features appears on more than 50% of the tested sessions, more 80% of the c-features do. The robustness of c-features will be crucial for improved performance in cross-session/subject test, as we will show in the next section.

**Figure 13:**
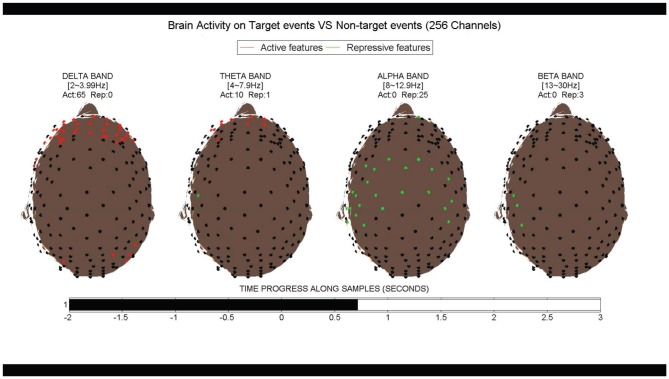
Movie for the robust discriminant space time-frequency features. The movie is aimed to show the most robust discriminant space time-frequency features in a RSVP task. Features here appear as top 10000 most discriminant features on more than 5 of the 10 tested sessions. The screen shot shows the frame at around 0.7 s, when alpha repression and delta band boost occur. Please visit http://compgenomics.cbi.utsa.edu/rsvp/index.html for the complete movie.

A 3D visualization system is also developed to show the most robust discriminant features that appear in the top 10000 discriminant features on more than 5 of the 10 tested sessions. Since these features are more consistent among multiple sessions, they are robust and should reflect the common brain response to the RSVP experiment. To reveal the most comprehensive information, we depicted the location, time, and frequency of the discriminant features in the movie. A sample screen shot is shown in Fig. 13 and the complete movie can be accessed from the project website http://compgenomics.cbi.utsa.edu/rsvp/index.html.

### Test on cross-sessions and cross-subjects

In real applications, training sessions are not always available for tested subjects. It is important to compare the robustness of LDA and cLDA on cross-session or cross-subject test. Since the data we adopted consists of 10 EEG sessions from 5 subjects (2 sessions per subject), we tested all the possible cross-session and cross-subject paired combinations, which include 20 pair-wise cross-session tests and 80 pair-wise cross-subject tests. In each possible combination pair, one session is used for training, while the other is for testing.

**Figure 14:**
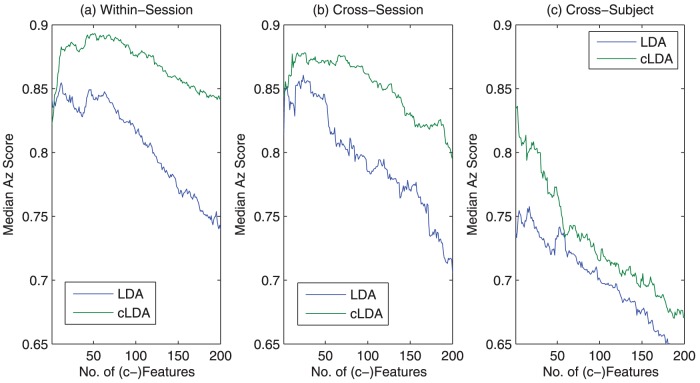
Comparison of cLDA and LDA in cross-session/subject tests. As expected, the performance of cLDA and LDA degrades when moving from within-session tests to cross-session tests, and then to cross-subject tests. However, regardless of the different number of c-features selected, cLDA performs better than LDA in all situations.

The median Az score performances are plotted in Fig. 14. As expected, the performance of cLDA and LDA degrades when moving from within-session test to cross-session tests, and then to cross-subject tests. However, regardless the different number of (c-)features selected, cLDA always performs better than LDA mainly due to better robustness of c-features. The best median performances are also summarized in Table 4. The robustness of c-features are apparently more advantageous when applied to cross-subject test, where cLDA achieves 0.08 increase in Az score over the within-session test as opposed to 0.04 improvement by LDA.

**Table 4:** Comparison of cLDA and LDA in cross-session/subject test.

Best Median Az Score	Within-Session	Cross-Session	Cross-Subject
LDA	0.85	0.86	0.76
cLDA	0.89	0.88	0.84

It is apparent that the performance of LDA was significantly degraded in the cross-subject test due to the lack of robustness in its top features.

### Test on human misclassified epoches

An important aspect of BCI systems is to assist human decision by identifying potential misclassified epoches. Given the experimental setting, it is impossible to time lock a misclassified non-target image clip. Instead, we examine whether it is possible to identify misclassified target image clips by test subjects.

**Figure 15:**
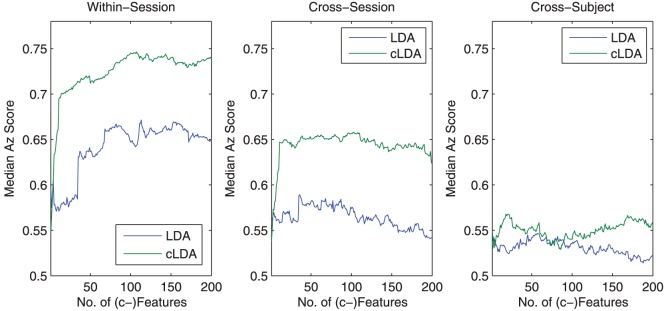
Identification of human error in classifying target image clips. The figure shows the classification performance of cLDA and LDA to identify human errors in classifying target image clips. cLDA can be better identify human errors especially for the within-session training data (Az score 0.75).

Once again, we used the within-session, cross-session, and cross-subject EEG data to perform the test but consider only the epochs that contain human misclassified target image clips as the positive epochs together with an equal number of non-target image epochs. The classification result is shown in Fig. 15. It can been clearly seen from the figure that both cLDA than LDA can identify the human incorrectly classified target image clips with good performance, especially for within-session tests. The task in cross-subject tests are apparently more difficult and neither can provide much improvement over the random decision. (Fig. 15-(b,c)). Between the two classifiers, cLDA clearly outperforms LDA with an Az score 0.74 vs 0.67 for the within-session test and 0.65 vs 0.59 for the cross session test (Fig. 15-(a)).

**Figure 16:**
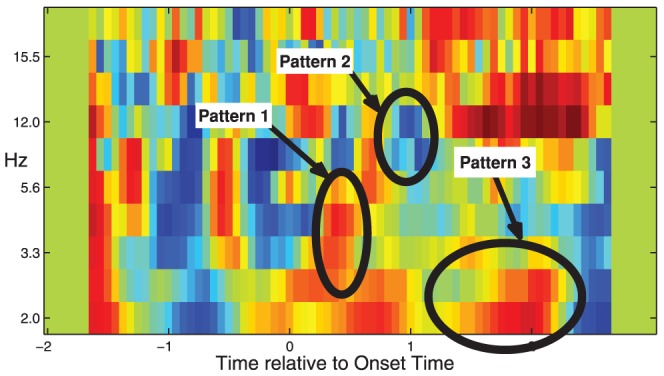
differences of features when correctly identifying and missing target images. The figure shows the power difference between subjects that correctly identified image clips and that missed ones. Compared with those that missed target clips, the subjects that correctly identified target image clips have stronger brain activities in pattern 1 and 3 but weaker activities for 3 patterns; this result is consistent with the difference in EEG patterns between target and non-target images and indicates that the subject's brain activities when missing a target image are the same as when seeing no target.

To further investigate which EEG features are related to correct and erroneous decisions, the feature patterns of subjects that correctly identified target images were compared with those that missed targets. The result is shown in Fig. 16. It can be seen that all the previous identified three discriminant patterns are also prevalent in this case. Particularly, subjects tend to exhibit stronger brain activities for pattern 1 and 3 and weaker activities around the three patterns when correctly identifying a target that those when missing a target. Interestingly, these patterns are consistent with the difference between target vs. non-target images (shown in Fig. 3), indicating that the subject's brain responses when missing a target image are the same as when seeing no target. The natural conclusion that can be drawn from this result is that the subjects that missed target images did not actually see the targets.

## Conclusions and Discussion

In this paper, the problem of automatic characterization and detection of target images in an image RSVP task based on EEG data is considered. The major contributions include:

- To characterize EEG recordings during an image RSVP event, we conducted time-frequency analysis and systematically identified the discriminant ERP features. A set of activated and repressed time-frequency features are identified. They represent three major distinct patterns within different time periods and frequency bands. A 3-D visualization system was developed for display of these features.
- We proposed a more robust cLDA classification algorithm for image classification. cLDA effectively combines correlated time-frequency features into uncorrelated c-features. We showed that cLDA outperforms LDA based on top features in within-session tests, cross-session tests, and cross-subject tests, regardless of the number of c-features used. Due to the robustness of c-features, cLDA performs better than LDA on cross-subject tests. We also showed that cLDA can identify human errors in classifying target image clips from EEG recordings, indicating its potential application in correcting human decisions based on EEG data.

Limited by the setting of the RSVP experiment, the extension of the proposed approach to multi-class detection is not straightforward. One existing approach that may tackle this issue is the “fern” based method [17], [18], which relies on an idea to eliminate the need to rank the discriminate features by grouping features as a “fern”. Investigation of such extensions would be important and especially valuable for its practical applications.

This work addresses the scenarios defined by the visual odd-ball paradigm, where the event timing information is available. However, in real application, due to practical limitations, stimulus onset timing may not be obtainable. Therefore, extension of the proposed c-feature to handle non-time-locked events will be of particular practical interest. It is worth mentioning that, the independent c-features we constructed not only retain the most useful discriminant information but are also shown to be more robust across multiple sessions/subjects. Given the potentially considerable increase in computation due to the additional need to infer the stimulus' onset time, using this compact and robust feature representation could be the key to the successful classification of non-time-locked events.

### Data and MATLAB code

The data and MATLAB code are available for download at http://compgenomics.cbi.utsa.edu/rsvp/index.html.

## References

[1] Wolpaw J, Birbaumer N, Heetderks W, McFarland D, Peckham P, et al (2000) Brain-computer interface technology: a review of the first international meeting. Rehabilitation Engineering, IEEE Transactions on 8: 164–173. 10.1109/tre.2000.847807 10896178

[2] Sajda P, Gerson A, Philiastides M, Parra L Single-trial analysis of eeg during rapid visual discrimination: Enabling cortically-coupled computer vision. Towards brain-computer interfacing.

[3] Bigdely-Shamlo N, Vankov A, Ramirez R, Makeig S (2008) Brain activity-based image classification from rapid serial visual presentation. Neural Systems and Rehabilitation Engineering, IEEE Transactions on 16: 432–441. 10.1109/TNSRE.2008.2003381 18990647

[4] Hild K, Pavel M, Erdogmus D, Mathan S (2009) Enhancing target detection using a hybrid humancomputer system. In: Signals, Systems and Computers, 2009 Conference Record of the Forty-Third Asilomar Conference on. IEEE, pp. 51–54.

[5] Eriksen C, Spencer T (1969) Rate of information processing in visual perception: Some results and methodological considerations. Journal of Experimental Psychology 79: 1. 10.1037/h0026873 5779623

[6] Squires N, Squires K, Hillyard S (1975) Two varieties of long-latency positive waves evoked by unpredictable auditory stimuli in man. Electroencephalography and clinical Neurophysiology 38: 387–401. 46819 10.1016/0013-4694(75)90263-1

[7] Kisley M, Cornwell Z (2006) Gamma and beta neural activity evoked during a sensory gating paradigm: effects of auditory, somatosensory and cross-modal stimulation. Clinical neurophysiology 117: 2549–2563. 17008125 10.1016/j.clinph.2006.08.003 PMC1773003

[8] Kirmizi-Alsan E, Bayraktaroglu Z, Gurvit H, Keskin Y, Emre M, et al (2006) Comparative analysis of event-related potentials during go/nogo and cpt: Decomposition of electrophysiological markers of response inhibition and sustained attention. Brain research 1104: 114–128. 16824492 10.1016/j.brainres.2006.03.010

[9] Fisher R (1936) The use of multiple measurements in taxonomic problems. Annals of Human Genetics 7: 179–188.

[10] Tolo¸si L, Lengauer T (2011) Classification with correlated features: unreliability of feature ranking and solutions. Bioinformatics 27: 1986. 21576180 10.1093/bioinformatics/btr300

[11] Delorme A, Makeig S (2004) Eeglab: an open source toolbox for analysis of single-trial eeg dynamics including independent component analysis. Journal of neuroscience methods 134: 9–21. 15102499 10.1016/j.jneumeth.2003.10.009

[12] Niedermeyer E, Da Silva F (2005) Electroencephalography: basic principles, clinical applications, and related fields. Lippincott Williams & Wilkins.

[13] Makeig S (1993) Auditory event-related dynamics of the eeg spectrum and effects of exposure to tones. Electroencephalography and clinical neurophysiology 86: 283–293. 7682932 10.1016/0013-4694(93)90110-h

[14] Fawcett T (2006) An introduction to roc analysis. Pattern recognition letters 27: 861–874.

[15] Park M, Hastie T, Tibshirani R (2007) Averaged gene expressions for regression. Biostatistics 8: 212. 16698769 10.1093/biostatistics/kxl002

[16] Cortes C, Vapnik V (1995) Support-vector networks. Machine learning 20: 273–297.

[17] Ozuysal M, Calonder M, Lepetit V, Fua P (2010) Fast keypoint recognition using random ferns. Pattern Analysis and Machine Intelligence, IEEE Transactions on 32: 448–461. 10.1109/TPAMI.2009.23 20075471

[18] Ozuysal M, Fua P, Lepetit V (2007) Fast keypoint recognition in ten lines of code. In: Computer Vision and Pattern Recognition, 2007. CVPR'07. IEEE Conference on. Ieee, pp. 1–8.

